# PReS-FINAL-2132: Defining criteria for high disease activity in juvenile idiopathic arthritis: definition and validation of juvenile arthritis disease activity score cutoffs

**DOI:** 10.1186/1546-0096-11-S2-P145

**Published:** 2013-12-05

**Authors:** A Consolaro, S Verazza, MC Gallo, G Bracciolini, G Negro, A Frisina, N Ruperto, A Martini, A Ravelli

**Affiliations:** 1 Pediatria II, Istituto Giannina Gaslini, Genova, Italy; 2 University of Genova, Genova, Italy

## Introduction

Assessment of disease activity is a fundamental component of the clinical evaluation of children with juvenile idiopathic arthritis (JIA) because persistently active disease plays a major role in causing joint damage and physical functional disability. Furthermore, measurement of the level of disease activity over time is important in monitoring the disease course and in assessing the effectiveness of therapeutic interventions.

## Objectives

To determine cutoff values for defining the state of high disease activity (HDA) in juvenile idiopathic arthritis (JIA) using the Juvenile Arthritis Disease Activity Score (JADAS).

## Methods

For the selection of cutoff values, data from a clinical database including 609 patients were used. Optimal cutoffs were determined against external criteria by calculating the 25th percentile of cumulative score distribution and through receiver operating characteristic curve analysis. External criteria were based on the therapeutic decision made by the attending physician at the time of the visit. The choice of cutoffs was made based on clinical and statistical grounds. Cross-validation was performed using 5 JIA patient samples that included a total of 1,421 patients, and was based on assessment of construct, discriminant, and predictive validity.

## Results

The best performance in selecting the cutoffs was provided by the 25th percentile approach. The final JADAS cutoff values were the following: 7 and 11 for JADAS27 in oligoarthritis and polyarthritis, respectively; 8 and 12 for both JADAS10 and JADAS71 in oligoarthritis and polyarthritis, respectively. In cross-validation analyses, the cutoff values revealed strong ability to discriminate between different levels of ACR Pedi response in 2 clinical trials and revealed good concordance with the subjective evaluations of the physicians and the parents. Furthermore, they proved able to predict a worse functional and radiographic outcome.

## Conclusion

Cutoff values for classifying HDA in JIA using the JADAS were developed. In cross-validation analyses, they proved to have good construct and discriminant validity and ability to predict disease outcome.

## Disclosure of interest

None declared.

